# Climate Signatures on Lake And Wetland Size Distributions in Arctic Deltas

**DOI:** 10.1029/2021GL094437

**Published:** 2021-10-19

**Authors:** Lawrence Vulis, Alejandro Tejedor, Ilya Zaliapin, Joel C. Rowland, Efi Foufoula‐Georgiou

**Affiliations:** ^1^ Department of Civil and Environmental Engineering University of California Irvine Irvine CA USA; ^2^ Department of Science and Engineering Sorbonne University Abu Dhabi Abu Dhabi United Arab Emirates; ^3^ Department of Mathematics and Statistics University of Nevada Reno Reno NV USA; ^4^ Earth and Environmental Sciences Division Los Alamos National Laboratory Los Alamos NM USA; ^5^ Department of Earth System Science University of California Irvine Irvine CA USA

**Keywords:** arctic, thermokarst lakes, remote sensing, wetlands, permafrost, deltas

## Abstract

Understanding how thermokarst lakes on arctic river deltas will respond to rapid warming is critical for projecting how carbon storage and fluxes will change in those vulnerable environments. Yet, this understanding is currently limited partly due to the complexity of disentangling significant interannual variability from the longer‐term surface water signatures on the landscape, using the short summertime window of optical spaceborne observations. Here, we rigorously separate perennial lakes from ephemeral wetlands on 12 arctic deltas and report distinct size distributions and climate trends for the two waterbodies. Namely, we find a lognormal distribution for lakes and a power‐law distribution for wetlands, consistent with a simple proportionate growth model and inundated topography, respectively. Furthermore, while no trend with temperature is found for wetlands, a statistically significant decreasing trend of mean lake size with warmer temperatures is found, attributed to colder deltas having deeper and thicker permafrost preserving larger lakes.

## Key Points

- Lake areas in arctic deltas exhibit a lognormal distribution associated with a simple mechanistic growth process
- Wetland areas exhibit a power law distribution consistent with inundated topography
- Colder arctic deltas have larger average lake sizes, likely due to thicker permafrost restricting sub‐lake hydrologic connectivity

## 1 Introduction

Coastal river deltas are landscapes at significant risk from sea level rise and sediment deprivation (Nienhuis et al., 2020; Syvitski et al., 2009). Arctic deltas are likely more vulnerable than their temperate counterparts due to the presence of thermokarst lakes in permafrost, which are sensitive to rapid Arctic warming (Emmerton et al., 2007; Piliouras & Rowland, 2020; Walker, 1998). Pan‐arctic thermokarst lake coverage is responding to warmer temperatures in complex ways, as temperature‐driven ground ice loss drives lake growth through retrogressive thaw slumping along lake shorelines (Grosse et al., 2013) but also generates surface and subsurface hydrologic connectivity that can cause lake drainage (Grosse et al., 2013; Jones et al., 2020; Rowland et al., 2011; Yoshikawa & Hinzman, 2003). Observed changes in lake area over the last 50 years have shown both positive and negative trends depending on local hydrology, climate, permafrost zonation, ice content, landscape age, and geomorphic setting (Arp et al., 2011; Chen et al., 2012; Jones et al., 2011; Nitze et al., 2018; Plug et al., 2008; Smith et al., 2005). Irrespective of whether lake coverage is expanding or decreasing, the reorganization of thermokarst lake cover will have significant implications for polar atmospheric carbon fluxes (Engram et al., 2020; Grosse et al., 2013; Petrescu et al., 2010; Rowland et al., 2010; van Huissteden et al., 2011; Walter Anthony et al., 2018). Moreover, thermokarst lakes in deltas modulate the transport of riverine freshwater, sediment, and nutrient fluxes to the Arctic ocean, by trapping and holding sediment (Marsh et al., 1999; Piliouras & Rowland, 2020) and modifying the residence times and pathways of nutrient transport through the delta (Cunada et al., 2021; Emmerton et al., 2007; Lesack & Marsh, 2010; Squires et al., 2009; Tank et al., 2009). Therefore, changing deltaic lake coverage and its spatial distribution will also alter the timing and magnitudes of riverine fluxes to the Arctic Ocean, which has broader implications for near‐shore circulation and ecosystem productivity (Lique et al., 2016).

**Figure 1:**
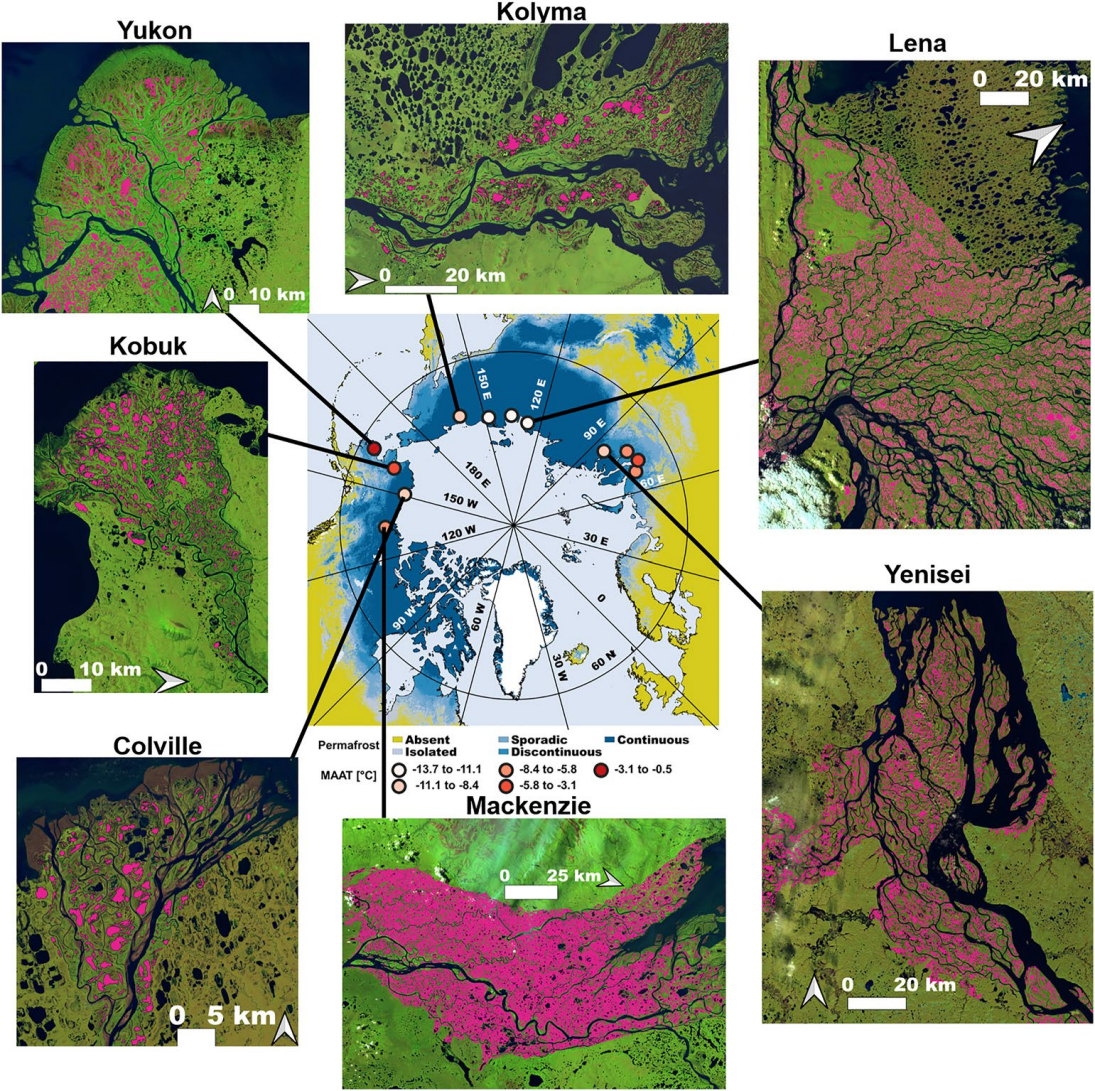
Arctic deltas examined in this study. Twelve arctic deltas were examined along a range of Mean Annual Air Temperature (MAAT) and ice content. The central map shows delta locations (circles), colored by 2000–2016 mean MAAT, estimated from the Arctic Systems Reanalysis V2 (Bromwich et al., 2018), and underlain by Arctic permafrost zonation (Obu et al., 2019). Summertime Landsat‐8 scenes of seven out of the 12 deltas are shown with waterbodies identified from a single July Global Surface Water mask (Pekel et al., 2016) colored in pink.

We hypothesize that lake size variability and spatial arrangement across arctic deltas (Figure 1) may encode information on climate influence in permafrost environments, akin to how channel network structure is a signature of the riverine, tidal, and fluvial fluxes, which shape temperate deltas (Nienhuis et al., 2016, 2018; Tejedor et al., 2015a, 2015b, 2016, 2017; see also Seybold et al., 2017; Zanardo et al., 2013 for the signature of climate in fluvial networks). In particular, we hypothesize that two primary drivers of lake size variability across deltas are ice content and climate and test this hypothesis quantitatively. Physically we expect that colder deltas have thicker permafrost, which is able to support larger lakes by preventing connection to the sub‐permafrost groundwater table that can lead to eventual lake drainage (Grosse et al., 2013; Walvoord & Kurylyk, 2016; Yoshikawa & Hinzman, 2003) or diminished lake growth rates. We also expect that deltas with greater soil ice fraction will have larger lakes as soil ice acts as a subsurface hydraulic barrier, while soil ice melt induces subsidence and therefore lake growth. The hypothesized relationships between lake size and ice content or temperature would be useful for constraining physical models and predicting the future of arctic delta morphology in a warmer climate.

However, a challenge in assessing the climatic signature on thermokarst lake sizes is the significant interannual (Grosse et al., 2013; Rey et al., 2019) and seasonal variability (Chen et al., 2012, 2013; Cooley et al., 2019; Vulis et al., 2020) in lake area, which makes it difficult to distinguish perennial waterbodies (lakes) from ephemerally inundated depressions (wetlands) using the short summertime window of available spaceborne observations. In particular, seasonal water may inundate ephemeral wetlands, which would be misidentified as perennially inundated lakes from remote‐sensing imagery. The processes underlying ephemeral wetland versus perennial lake formation are distinct, as lakes are the result of thermokarst‐driven growth and evolution (Grosse et al., 2013), while wetlands are the result of hydrologic variability (Le & Kumar, 2014), and as defined in this study, only seasonally inundated. These ephemerally inundated wetlands likely have less significant thermal impacts on the landscape than lakes, and are thus expected to lack a relationship with delta climate. In the remainder of this study, we present a methodology to mine the historical Landsat imagery record to distinguish lakes and wetlands, and then characterize their respective size distributions as well as their potential dependence with climate.

## 2 Study Sites, Data, and Lake and Wetland Extraction

Lake and wetland size distributions on 12 arctic deltas characterized by a range of air temperature and ice content across Siberia (Indigirka, Kolyma, Lena, Nadym, Ob, Pur, Yana, and Yenisei), Canada (Mackenzie), and Alaska (Colville, Kobuk, and Yukon) were examined (Figure 1). The deltas include those formed by the six arctic rivers with the greatest discharge and other major rivers along the Siberian and Alaskan coastlines. Lakes and wetlands were extracted over the subaerial portion of each delta, which was delineated using Google Earth. Delta Mean Annual Air Temperature (MAAT) was obtained from 2000 to 2016 using the 15 km spatial resolution Arctic Systems Reanalysis V2 (Bromwich et al., 2018). Delta soil ice content was estimated from a 12.5 ‐km spatial resolution ice classification map (Brown et al., 1997).

To distinguish between hydrologically perennial lakes and ephemeral wetlands, we utilized the spatiotemporal interannual variability of water coverage over each delta from 1999 to 2018. We used the Landsat‐derived, 30 ‐m spatial resolution Global Surface Water (GSW) data set, which provides monthly composited water masks from March 1984 to December 2018 that classify the landscape into 30‐m pixels that are land, water, or no data (i.e., unable to classify due to cloud cover, Landsat‐7 striping, or snow and ice cover) (Pekel et al., 2016). Due to sparse data availability prior to 1999 on most deltas, we only analyzed the period from 1999 to 2018, and to remove the effect of significant snowmelt and spring time flooding, we restricted our analysis to July water masks similar to other studies (Muster et al., 2019; Nitze et al., 2018). We only examined the subaerial portion of each delta, manually delineated using Google Earth.

**Figure 2:**
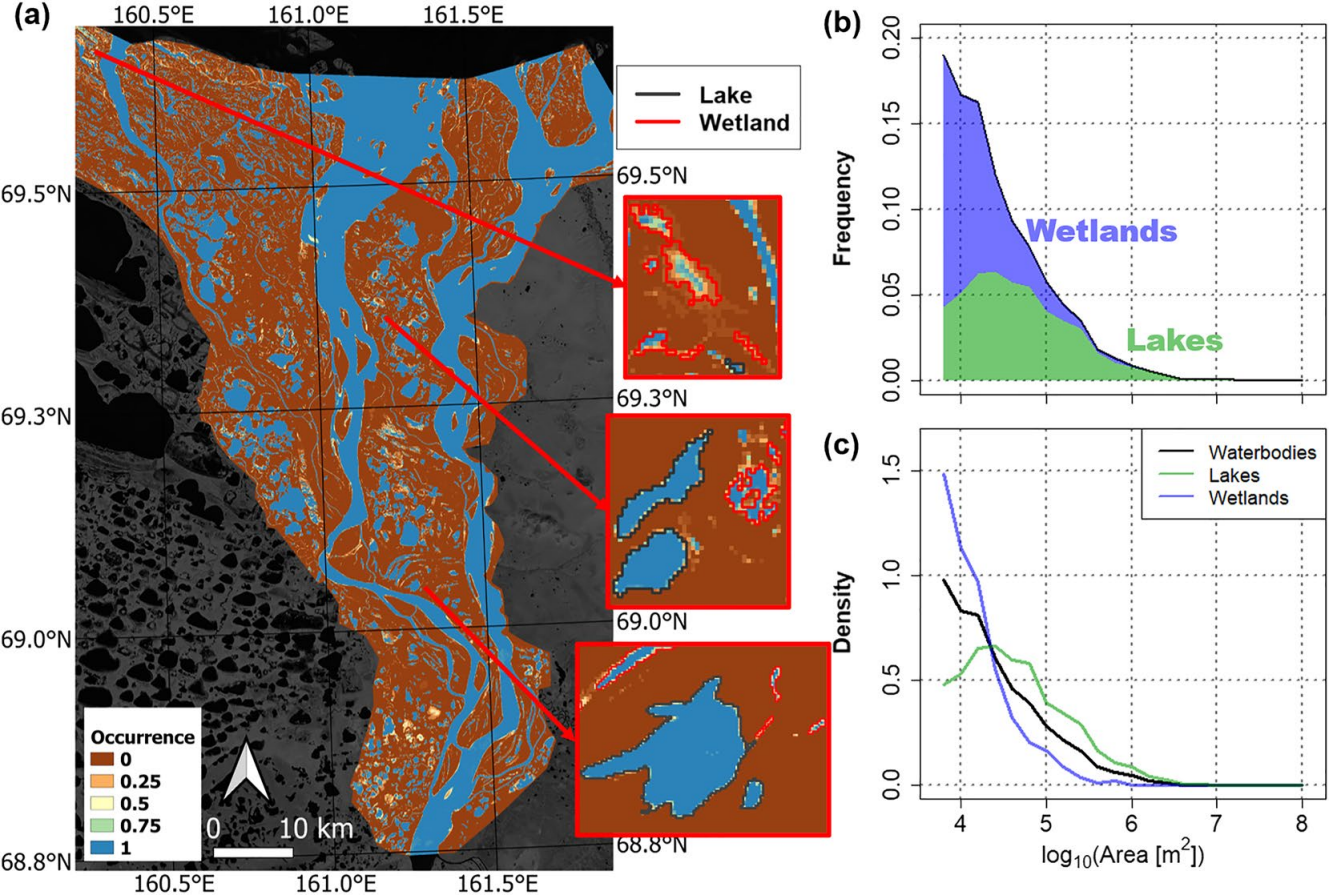
Example of waterbody classification procedure on Kolyma Delta. The waterbody classification procedure, which marks waterbodies as either perennial lakes or ephemeral wetlands based on their July occurrence index, and the resulting size distribution. (a) July pixel water occurrence $w_{i}$ over the Kolyma delta from 1999 to 2018. Brown indicates land pixels $\left(w_{i}=0\right)$ and blue indicates perennially inundated water pixels $\left(w_{i}=1\right)$, with colors in between indicating water pixels indicated only a fraction of the time. (b) The histogram of waterbody sizes is partitioned into the relative fraction of lakes (green) and wetlands (blue) at an occurrence index threshold θ=0.85. (c) The probability density function (PDF) of lake sizes in green and wetland sizes in blue, compared with waterbody sizes in black.

**Figure 3:**
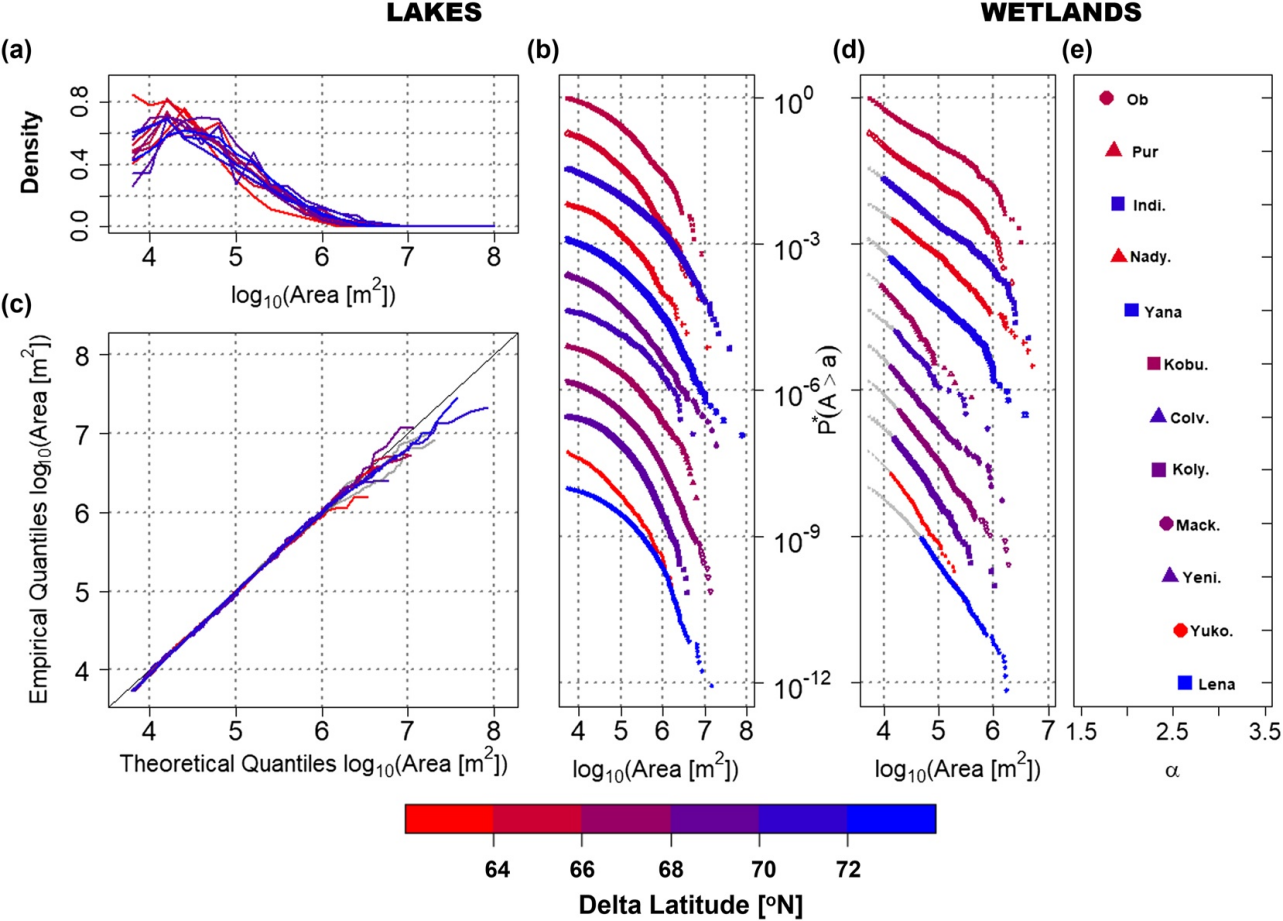
Size distributions of lakes and wetlands extracted at occurrence index threshold θ=0.85. (a) Lake size probability density functions (PDFs) for the 12 deltas, (b) lake size exceedance probabilities, and (c) quantile‐quantile plots of the lognormal with truncation from below at the minimum lake size (5,400 m^2^) fitted to the lake size distribution. In (b) fitted lognormal (LN) distributions whose fit to data is rejected at the 5% significance level (Kolmogorov‐Smirnov (KS) test) are in gray. (d) Wetland size exceedance probability, (e) fitted PDF power law exponent, α, of all 12 deltas. The exceedance probabilities in (b) and (d) are rescaled by a factor τ, that is, $P^{*}=P\tau$, for visual display and comparison of the differences between lake and wetland size distributions on each delta. The distributions are ordered by increasing values of α to highlight the range of observed α. For each delta, power laws are fit to the colored points in (d) above the minimum wetland size, *x*_*0*_, which was optimally determined using the procedure of Clauset et al. (2009).

**Figure 4:**
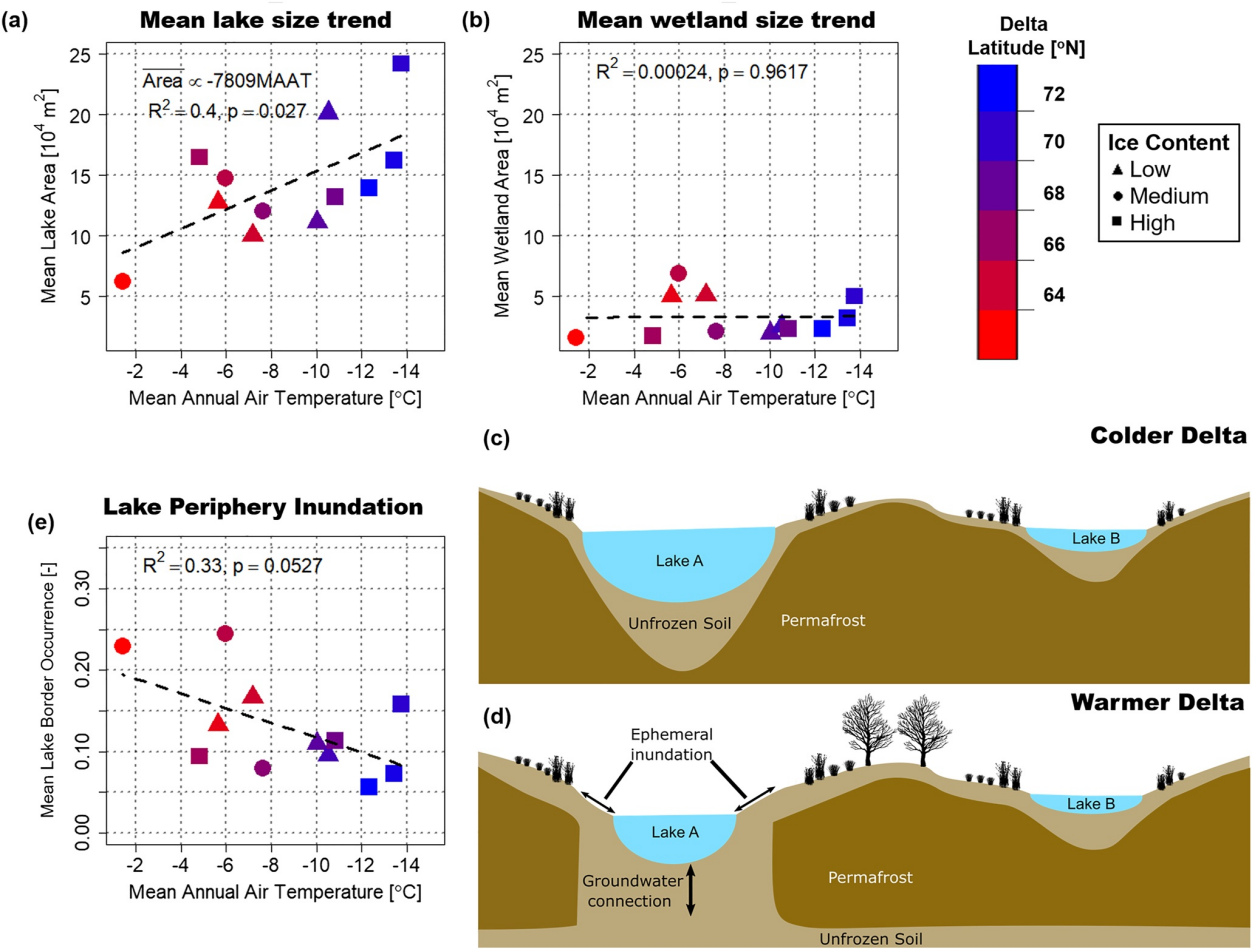
Lake and wetland size climate trends. (a) Scatterplot between mean lake size and Mean Annual Air Temperature (MAAT) showing a significant relationship between the two, with bootstrap *p* = 0.0264 and Spearman rank correlation −0.5 (*p* = 0.1038). A significant trend between the 90th percentile of lake sizes and MAAT (*p* =  0.041, bootstrap *p* = 0.0366, and *R* ^2^ =  0.36) was also found (not shown). (b) Scatterplot between mean wetland size and MAAT showing the lack of a significant relationship. (c and d) The relationship between lake size and MAAT is attributed to colder deltas having thicker permafrost, which prevents lakes from connecting to the sub‐permafrost aquifer. In warmer deltas, connection to the sub‐permafrost aquifer leads to greater lake level change over the summer, driving increased variability in inundation along the peripheries of lakes, and diminishing rates of thermally driven lateral expansion. (e) Scatterplot between the fraction of the periphery of large lakes that remains water on average over the period of record and MAAT shows a weak (i.e., *p* = 0.0527, bootstrap *p* = 0.0551) linear relationship, supporting the proposed mechanism in (c and d).

To identify and separate lakes from wetlands, we first computed for every pixel *i* the July “water pixel occurrence,” $w_{i}$, as the fraction of Julys from 1999 to 2018 for which the pixel was classified as water, discarding no‐data pixels (Figure 2a). The water pixel occurrence $w_{i}$ can take values from 0 to 1, with $w_{i}=1$ if and only if the pixel was classified as water for the whole record, and $w_{i}=0$ if and only if the pixel was classified as land for the whole record. Second, we identified a reference year, $y^{*}$, with water coverage on the subaerial delta closest to that of the temporal average over the 20‐year period of record and sufficient data quality (i.e., greater than 99% pixels classified as land or water and no significant georeferencing (collocation) errors) and used this year to identify individual waterbodies using 8‐neighbor connected component analysis (see Supporting Information S1, Figures S1 to S3 for details on selection of $y^{*}$). Third, we classified the waterbodies identified in year $y^{*}$ into lakes and wetlands using the water pixel occurrence, $w_{i}$. For each waterbody, $O_{k}^{y^{*}}$, we computed the “occurrence index” $B_{k}$ as the mean of $w_{i}$ for all pixels i within $O_{k}^{y^{*}}$, which corresponds to the fraction of pixels within the waterbody that were on average occupied by water over the 20 years (Julys) of record. A waterbody was then classified as a lake if $B_{k}$ exceeded a threshold value θ and as a wetland if $B_{k}$ was less than θ. We evaluated the results over a range of θ values, from θ=0.80 to θ=0.90, to account for differences in the flooding regime across different deltas and to test the robustness of our results (Tables S1–S3 and Figures S4 and S5 in the Supporting Information S1). The lake and wetland size distributions shown in Figures 3 and 4 are extracted at a threshold value of θ=0.85. Only waterbodies at least 5,400 m^2^ (i.e., six pixels) in size were included in our analysis to reduce estimation errors at small areas. We tested the robustness of our methodology by performing a duplication, wherein we selected an alternative reference year, $y_{alt}^{*}$, with similar water coverage and data quality to extract waterbody extents and repeated the analysis (Supporting Information S1, Table S4, and Figures S4 and S5). All analyses were performed in R using geospatial and image processing packages (Gillespie, 2015; Hijmans, 2020; Pau et al., 2010; Pebesma, 2018, 2020).

## 3 Lake Size Distributions And a Proportionate Growth Model

From a simple thermodynamical perspective, thermokarst lakes are thermal reservoirs, which interact with their surroundings through heat exchange. In particular, unfrozen lake waters are net heat sources, thawing the surrounding ice‐rich soil, which leads to lake basin expansion (Grosse et al., 2013). As larger lakes have a larger thermal inertia, they remain unfrozen for longer periods (Grosse et al., 2013) and maintain larger lake to soil temperature gradients, which enables them to grow at faster rates. Thus, based on this simple thermodynamical argument, and on field observations (Jones et al., 2011), we can postulate that thermokarst lake growth is compatible with a stochastic proportionate growth model (Crow & Shimizu, 1988; Mitzenmacher, 2004) (i.e., growth rate proportional to lake size), where stochasticity arises from the variability of soil properties that modulate growth. A key property of this general class of proportionate growth models is that they generate objects (in our case lakes) with sizes obeying a lognormal (LN) distribution (Supporting Information S1) (Crow & Shimizu, 1988). Thus, our expectation based on simple physical arguments is that arctic deltas should universally exhibit lakes whose sizes are lognormally distributed. In particular, since we only observe lake sizes above 5,400 m^2^ (six pixels), we expect lake sizes to follow a truncated lognormal distribution (Equation 1):

(1) $$f_{x}\left(x;\;\nu ,\;\beta^{2}\right)=\{\begin{array}{l} \;0\;\;\;\text{for}\;x<x_{\text{min}}\;\\ \frac{\frac{1}{x\beta \sqrt{2\pi }}\;e^{\frac{-{\left(\ln\left(x\right)-\nu \right)}^{2}}{2\beta^{2}}}}{\left[1-\Phi \left(\frac{\ln\left(x_{\text{min}}\right)-\nu }{\beta }\right)\right]}\;\text{for}\;x\geq x_{\text{min}}\; \end{array},\;$$

where Φ(·) is the cumulative distribution function (CDF) of a standard normal variable, ν is the scale parameter, β the shape parameter, and $x_{\text{min}}$ the minimum value at which the LN is observed, here 5,400 m^2^ (Clauset et al., 2009). When $x_{\text{min}}$ approaches zero, the denominator approaches unity and Equation 1 is simply the LN distribution.

Having separated lakes and wetlands based on the methodology outlined in Section 2, we examined the empirical probability density function (PDF) and exceedance probability of lake sizes (Figures 3a and 3b). As postulated, we found that the examined lake sizes can be accurately described by a truncated LN distribution for the whole range of lake sizes (spanning 3.5 orders of magnitude) in the 12 deltas under study (see Quantile‐Quantile (Q‐Q) plots in Figure 3b). The rigorous Lilliefors‐corrected Kolmogorov‐Smirnov (KS) test (Clauset et al., 2009) shows that for every delta, the fitted LN distribution could not be rejected at the 5% significance level within the range of thresholds θ utilized for the identification of lakes from the general waterbody population (Tables S1 to S3 in the Supporting Information S1). For most deltas, the LN fit could not be rejected over the entire range, but in several deltas the test outcome depended on the threshold, due to the fact that the hydrogeomorphological specificities of the different deltas can lead to potential suboptimal lake/wetland separation for certain threshold values and ranges of waterbody sizes. Furthermore, the robustness of the revealed universality of the LN distribution of lake sizes was confirmed by successfully testing that lake sizes are LN distributed when alternative years were used as reference to extract waterbodies (Table S4, Figure S4 in the Supporting Information S1). Previous empirical (suggesting different distributions for arctic waterbodies) (Muster et al., 2019) and theoretical (suggesting a proportionate growth model) (Victorov et al., 2019) studies have failed to demonstrate this universality because thermokarst lakes and wetlands were analyzed together (Table S5 and Figure S6 in the Supporting Information S1), and as we show in the next section, wetlands do exhibit a different distribution.

## 4 Wetland Size Distributions And an Inundated Topography Model

Arctic delta wetlands are, by definition, ephemeral waterbodies emerging on the delta top due to local ice/snow melt and riverine flooding. Therefore, wetland sizes are expected to be highly dependent on the seasonal delta hydrology, which controls overall delta wetness (hydrologic forcing), and delta topography; the topography in turn constitutes the spatial layout for inundation and controls both the emergence of disjoint wetlands and their sizes for a given forcing. The prevalence of power law distributions describing the sizes of waterbodies emerging from landscape inundation has been extensively documented (Bertassello et al., 2018; Cael & Seekell, 2016; Cael et al., 2015; Le & Kumar, 2014; Mandelbrot, 1982; Messager et al., 2016). For instance, recent analysis of the sizes of wetlands identified from inundating low‐relief topography and observed wetlands in the contiguous United States were found to exhibit power law distribution of areas consistent with inundated topography (Bertassello et al., 2018; Le & Kumar, 2014). Therefore, our hypothesis was that the Arctic delta wetlands will follow a similar distribution. The form of the power law PDF used in this study is given in Equation 2, where $x_{0}$ is the minimum size above which the power law is fit and α is the power law exponent (Clauset et al., 2009):

(2) $$f_{X}\left(x;\alpha \right)=\frac{\alpha -1}{x_{0}}\;{\left(\frac{x}{x_{0}}\right)}^{-\alpha },\;x>x_{0}$$

We observed that wetland size distributions in the 12 arctic deltas indeed show strong evidence of being power law distributed (log‐log linearity over two orders of magnitude in Figure 3d). Using the robust methodology of Clauset et al. (2009) for power law testing and fitting, we found that the power law hypothesis for wetland sizes could not be rejected at the 5% significance level with a Lilliefors‐corrected KS test for 11 out of 12 deltas (at θ=0.85, Table S1 in the Supporting Information S1). As with lakes, the power law distribution of wetland sizes is robust with respect to the threshold θ, which establishes the separation of waterbodies into lakes and wetlands (Tables S2 to S3 in the Supporting Information S1). Moreover, the robustness of our hypothesis was verified by extracting waterbodies and identifying wetlands in an alternative reference year, wherein again most deltas displayed power law wetland size distributions (Table S4, Figure S4 in the Supporting Information S1). The observed power law exponents range from 1.8 to 2.8 and are similar to what has been found for wetlands in the contiguous United States (Bertassello et al., 2018; Le & Kumar, 2014) and other waterbodies on multiple scales (Cael et al., 2015). The range in the observed exponents at different thresholds θ is attributed to the hydrogeomorphic variability within and across the deltas, and the imperfect separation between lakes and wetlands. We point out that the evaluation of alternative models such as the LN distribution cannot be performed on a statistical basis, for example, using the Akaike Information Criterion (Burnham & Anderson, 2004), due to the limited sample size (for more details see Supporting Information S1; Figure S8; Clauset et al., 2009). However, the observed power law exponents and the lack of interpretability of the alternative distribution (e.g., LN) parameters strongly suggest that the power law is a physically meaningful model to describe the wetland size distribution (Supporting Information S1).

Recent literature has hypothesized that lakes in the Arctic are consistent with landscape inundation mechanisms (Muster et al., 2019). This hypothesis was grounded on the finding that empirical statistics of waterbodies obey two relationships (a linear relationship between conditional mean and conditional variance and a hyperbolic relationship between conditional mean and conditional skewness), which are consistent with those arising from an inundation model experiment (Muster et al., 2019). However, as we show here (Supporting Information S1, Figure S9), these same relationships arise from a proportionate growth model and an LN distribution, cautioning their use for distinguishing between the power law and LN probability distributions and for making physical inferences.

## 5 Climate Trends

How thermokarst lake coverage on arctic deltas will respond to projected 21st century warming is a question of critical interest due to the impacts on methane emissions (Engram et al., 2020; Petrescu et al., 2010; van Huissteden et al., 2011), the release of old carbon (Grosse et al., 2013; Rowland et al., 2010), replumbing of surface‐subsurface hydrologic partitioning (Walvoord & Kurylyk, 2016), and changes in water and biogeochemical cycling to the ocean (Piliouras & Rowland, 2020; Piliouras et al., 2021). Discovering robust relationships between lake size distributions and climate variables such as temperature and soil ice content would provide valuable insight into the future of lake coverage on arctic deltas. Given the clear differences in lake versus wetland size distributions (Figure 3) and their associated generative processes, we reemphasize the hypothesis that only lake sizes should encode the signature of climate through temperature and ice content, while ephemeral wetlands should be agnostic to it.

We have tested this hypothesis by analyzing the relationships between mean lake and wetland size (areal extent) with respect to MAAT and soil ice content. The data suggest that the mean thermokarst lake size increases by 9·10^4^ m^2^, that is, doubling, over a 12°C decrease in the average 2000 to 2016 MAAT (Bromwich et al., 2018), indicating that colder deltas have significantly larger lakes on average (Figure 4a). Modern MAAT may not be representative of paleoclimatic temperature variability; however, mean lake size also has a significant linear relationship (*p* =  0.023, bootstrap p=0.023, and *R* ^2^ =  0.42) with delta apex latitude, which is a reasonable proxy for historical temperature differences between the deltas, strongly supporting a temperature to lake size relationship. Mean lake size also generally positively relates to soil ice content, as higher ice content on the delta may support lake growth due to greater settlement from ice melt (Grosse et al., 2013), with lower ice content associated with smaller lakes (Figure 4a). A similar trend between lake sizes and MAAT is observed when an alternative reference year is used to extract waterbodies in Supporting Information S1 (Figure S5a), supporting the robustness of this dependence. On the other hand, the data show no relationship between mean wetland size and MAAT (Figures 4b and S5b). Also expected, but confirmed, mixing the two waterbodies makes it hard to detect the climatic signal on the landscape. Indeed, a joint analysis reveals a nonsignificant relationship with MAAT (Figure S6d in the Supporting Information S1).

The observed relationship for mean lake size and MAAT is attributed to the greater capacity of colder deltas to support large lakes due to their presumably thicker and cooler permafrost, which prevents sub‐lake taliks from connecting to the sub‐permafrost groundwater table (Walvoord & Kurylyk, 2016). This connection in low relief deltaic environments would reduce lake level as river stage recedes through the summer, transitioning the margins of perennially inundated lakes to ephemerally inundated, thereby reducing lateral thermal fluxes from the lake to the surrounding permafrost, that is, diminishing lake growth and decreasing the observed size of perennially inundated lakes (Figures 4c and 4d). Such an effect would be clearest in large lakes that have deep taliks (Grosse et al., 2013), and indeed, we found that the peripheries of large lakes were inundated more often on average over the period of record on warmer deltas compared with colder deltas (see Figure 4e). Note that the fraction of the periphery that remains water (inundated) on average over the period of record was quantified as the mean $w_{i}$ of all pixels bordering each lake (in an 8‐neighbor sense), and the average value for the large lakes (defined as those with areas between 10^5^ and 10^6^ m^2^) is reported for each delta.

Such a relationship between MAAT and lake periphery inundation may also occur due to evapotranspiration rates being higher on warmer deltas, which leads to greater lake margin loss. However, we found that average June‐July precipitation minus evapotranspiration (P‐ET, i.e., the vertical hydrologic budget) (Bromwich et al., 2018) over the delta is uncorrelated with MAAT, and therefore P‐ET does not explain the relationship between delta temperature and how often lake peripheries are inundated (Figure S5d in the Supporting Information S1). This mechanism could be validated in future studies by imaging subsurface permafrost structure across the deltas, which has been done in other lake‐rich permafrost environments (Rey et al., 2019).

## 6 Perspectives and Conclusions

By harnessing more than 20 years of remote sensing data over the Arctic, we have developed a methodology to classify waterbodies, depending on their year‐to‐year variability as lakes (perennial) and wetlands (ephemeral). The statistical distributions of lake and wetland sizes are distinct and appear to be universal across arctic deltas, reflecting the respective underlying mechanisms driving the formation and evolution of those waterbodies. Specifically, it was found that thermokarst lake sizes obey a lognormal distribution, which can be interpreted as the emergent signature of the thermal mechanism driving lake formation and growth. On the other hand, wetland sizes may be described by a power law distribution, which is compatible with landscape inundation models relevant to ephemeral waterbodies (Bertassello et al., 2018; Le & Kumar, 2014). The difference between the underlying forming mechanisms leads also to different expectations with respect to possible relationships with climatic variables. Indeed, our results reveal a significant trend between mean lake size and mean annual air temperature, supporting the hypothesis that colder environments are able to grow and sustain larger thermokarst lakes, while no signature of climate is found in the mean wetland sizes. The power law exponents of the wetland size distributions were found to range between 1.8 and 2.8 (a smaller exponent indicates a thicker tail of the PDF) in line with what has been observed in other regions (Bertassello et al., 2018; Cael et al., 2015; Le & Kumar, 2014) and further analysis of high‐resolution topography is expected to provide additional insight on this range. The decreasing trend of mean lake size with warmer temperatures found here can form the basis for future lake area change projections; however, recognizing that the relationship from the 12 examined deltas, although statistically significant, explains only 40% of the variance and lake change may display significant spatial variability (Chen et al., 2012). These relationships provide some of the first quantification of climate influence on delta morphology along with other recent work on channel network structure (Lauzon et al., 2019; Piliouras et al., 2021). Spatially resolved permafrost depth and ground ice content on the deltas (Rey et al., 2019), as well as analysis of physically based models forced with different climate scenarios (Coon et al., 2019; Overeem et al., 2018) is needed to better understand cause‐and‐effect and derive relationships that can serve as the basis of projections of landscape change (e.g., increased water ephemerality under warming scenarios) and associated carbon cycle impacts in specific delta environments. Major arctic deltas store approximately 91 ± 39 Pg‐Carbon, potentially making them significant sources of future carbon emissions (Schuur et al., 2015), motivating the need for further study of the biogeochemical cycling in these landscapes.

## Supporting information

Supporting Information S1

## Data Availability

The Global Surface Water monthly water masks are available through Google Earth Engine (https://developers.google.com/earth-engine/datasets/catalog/JRC_GSW1_1_GlobalSurfaceWater?hl=en). The Arctic Systems Reanalysis V2 data are available from University Corporation for Atmospheric Research (UCAR) Research Data Archive (RDA) (https://rda.ucar.edu/datasets/ds631.1/). The ice content data are available from the National Snow and Ice Data Center (NSIDC) (https://nsidc.org/data/ggd318). Code to reproduce this analysis is available at Zenodo: https://doi.org/10.5281/zenodo.5504431.
