# Supplementary material for: Climate Signatures on Lake And Wetland Size Distributions in Arctic Deltas
Source: Geophys Res Lett. 2021 Oct 19;48(20):e2021GL094437. doi: 10.1029/2021GL094437 (PMC9285363; doi:10.1029/2021GL094437)
Supplement: Supplementary file 1 — Supporting Information S1 [file GRL-48-0-s001.pdf]

Climate signatures on lake and wetland size distributions in arctic deltas

Supplementary Material

Lawrence Vulis[1], Alejandro Tejedor[2,1], Ilya Zaliapin[3], Joel Rowland[4], and

Efi Foufoula-Georgiou[1,5]

[1]Department of Civil and Environmental Engineering, University of California Irvine, lvulis@uci.edu
[2]Department of Science and Engineering, Sorbonne University Abu Dhabi
[3]Department of Mathematics and Statistics, University of Nevada Reno
[4]Earth and Environmental Sciences Division, Los Alamos National Laboratory
[5]Department of Earth System Science, University of California Irvine

Table of Contents

I. Quality control of the Global Surface Water dataset 3

II. Hydrology of the deltas and choice of the year for waterbody mask extraction 5

III. Proportionate growth model 8

IV. Fitted distribution parameters and climate trends for lakes, wetlands, and waterbodies 9

V. Model identification for small samples of power-law distributed data. 16

VI. Relationships between the first three conditional moments 18

I. Quality control of the Global Surface Water dataset

Thorough quality control of the water masks is necessary to reduce uncertainty in the estimated pixel water occurrence w_i and therefore the waterbody classification scheme. In particular, misclassified or poorly classified masks, e.g. where land pixels are classified as water or vice-versa, particularly in the presence of abundant unresolved pixels (i.e. pixels unable to be classified as land or water due to cloud cover, Landsat 7 striping, or other issues), introduce errors into the estimate of w_i, which lead to waterbody misclassification. To address this, we performed the following quality control procedure consisting of a combination of quantitative rules and visual inspection on the GSW monthly water masks for all 12 deltas. First, for every delta we discarded from the analysis any mask over the period of record that had less than 10% of the study region resolved, as we observed misclassification errors for such poor-quality data. Second, we performed a visual inspection for significant misclassification errors, e.g. stripes of pixels classified as land or water or large swaths of the region appearing to be land only for a single year, and found only July 2016 on the Lena delta had to be discarded. Third, we identified and estimated mis-collocation errors in the GSW dataset of at least 1 pixel (30 meters) over the Yana delta from 2016 to 2018 and Lena delta from 2017 to 2018 relative to the masks from 1999 to 2015. These years were discarded from the computation of the July water pixel occurrence, w_i, but were used to estimate the average water cover since mis-collocation does not imply features were misclassified, only that their locations were shifted. No miscollocation on the order of one pixel (30-m) was observed on the other 10 deltas from 1999 to 2018. Note that the Pechora delta has not been considered in this work because of a large collocation error even in GSW v1.0 (i.e. years prior to 2016).

An example of the collocation errors is shown for the Yana delta, where waterbodies extracted from July 2018 are shifted to the north-west compared to waterbodies extracted from July 2011 (Figure S1). Due to interannual variability in surface water extent and a lack of ground control points, we were not able to compute the exact collocation error over the region and to correct the masks. Therefore, to estimate the magnitude of the miscollocation, we looked at the distribution of differences in waterbody centroids between different years, $(\Delta C_x, \Delta C_y)$. We found that the median of $(\Delta C_x, \Delta C_y) = (C_{x,2011} - C_{x,2018}, C_{y,2011} - C_{y,2018})$ was (29.24, -11.06) m, i.e. the median centroid difference between the two masks was approximately one pixel in the horizontal direction and a third of a pixel in the vertical. By examining the whole distribution of differences in waterbody centroids, we quantified that over 88% percent of waterbodies in 2018 were shifted to the southwest relative to the position of the same waterbodies in 2011 (i.e. over 88% of the centroids lay within the lower right quadrant of Figure S1b).

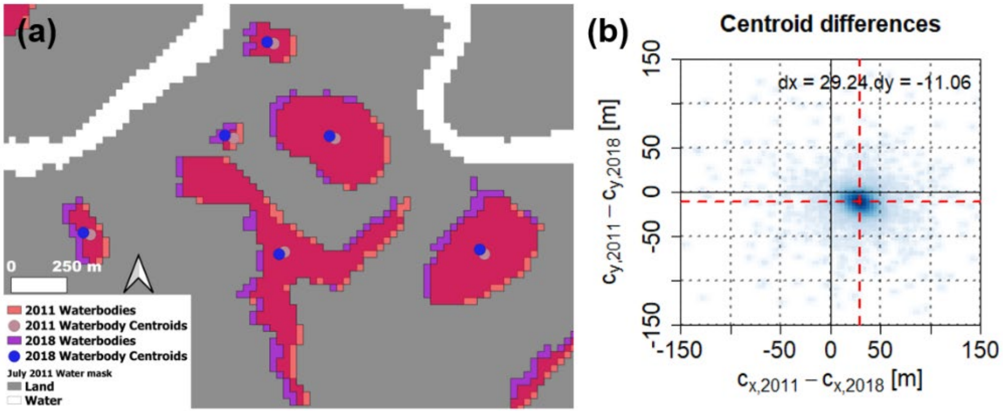

Figure S1. Collocation errors in the GSW dataset on the Yana delta. (a) Waterbodies from 2011 (red) and 2018 (purple) overlaid over the July 2011 water mask, with a clear offset between the two. The corresponding waterbody centroids are shown in brown and blue, respectively. (b) The distribution of centroid differences is shown with the median difference in each direction given by the red dashed line.

II. Hydrology of the deltas and choice of the year for waterbody mask extraction

To choose the reference year y^* in which to extract waterbody extents as objects and classify perennial lakes and ephemeral wetlands based on their year-to-year variability, we first computed for each delta and year the water cover, i.e. the fraction of valid (i.e. resolved as water or land) pixels that are classified as water over the subaerial delta, defining time series of July water cover from 1999 to 2018 (Figures S2 and S3). Then, we computed for each delta the average water cover over the period of record using the total number of valid pixels in each year as weights. Finally, y^* was chosen as the year with water cover closest to the average and at least 99% valid pixels. To test the robustness of the results, an alternative reference year, y^*_{alt} was also selected for each delta with a similar water cover to y^* and high data quality and the analysis repeated (Table S4 and Figures S4 and S5). To account for the heterogeneity in data quality across the range of analyzed systems, exceptions to these criteria had to be made for the Yukon, Lena, and Indigirka deltas. On the Yukon delta, the only two years satisfying the 99% valid pixel criterion were the 2008 and 2014, but these two are the wettest years on record, not years with typical hydrology. Therefore, 2017 and 2016 which had 98.7% and 98.9% valid pixels (slightly less than the 99% criterion), but close to average water cover were chosen as y^* and y^*_{alt}, respectively (Figure S2). On the Lena and Indigirka deltas only 2013 and 2016, respectively, had at least 99% valid pixels for the period of record. To perform the replication analysis, we relaxed the 99% valid pixels criterion to identify an alternative reference year y^*_{alt}. We found that 2007 had 98.5% valid pixels over the Lena delta and 98.7% valid pixels over the Indigirka delta, and therefore chose 2007 as y^*_{alt} for both deltas.

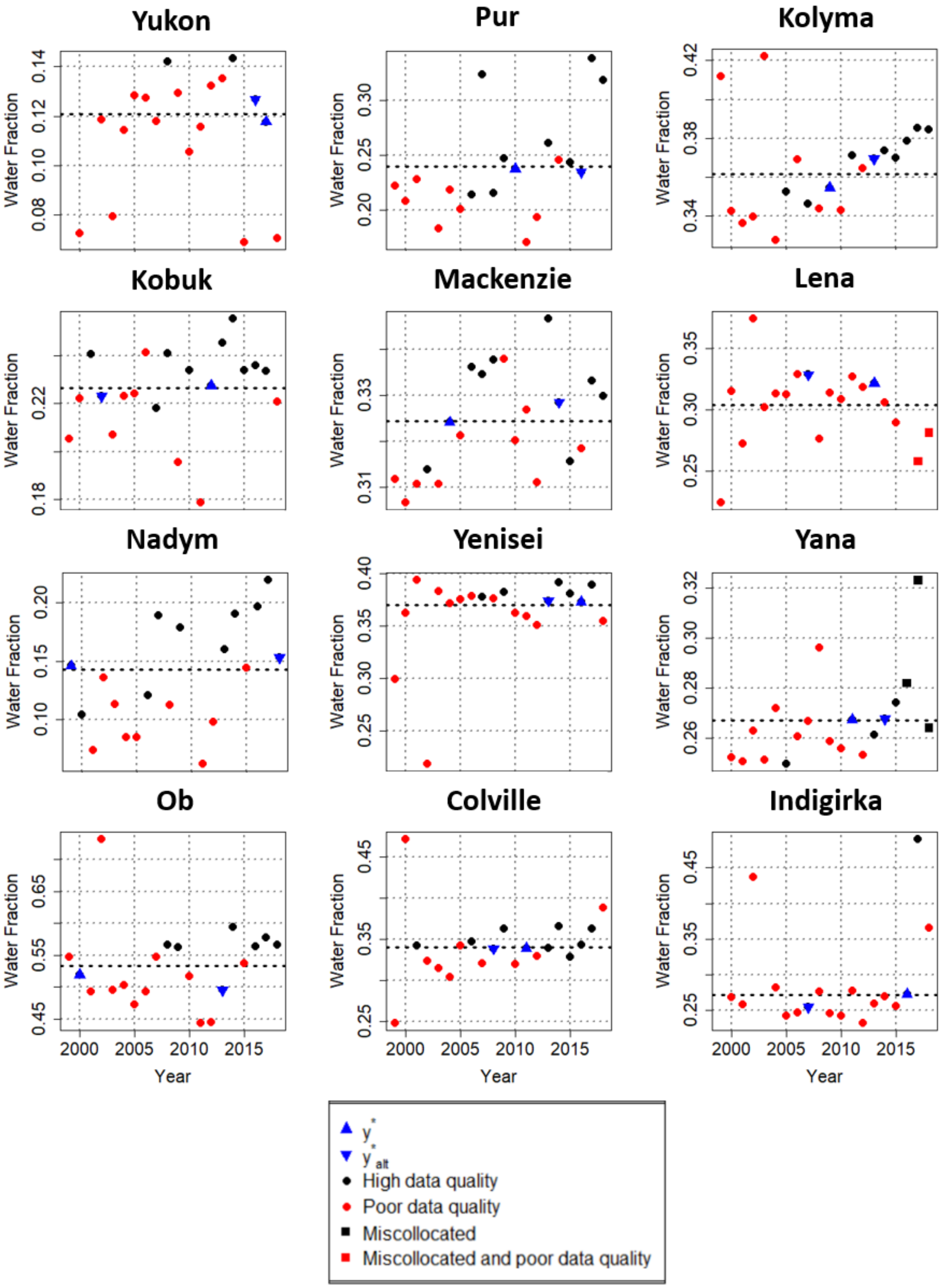

Figure S2. Surface water hydrology of arctic deltas. Time series of July water cover for every delta from 1999 to 2018. Years with at least 99% valid pixels are marked in black and years with less than 99% valid pixels in red, while years chosen for waterbody extraction are in blue triangles. Miscollocated years are shown with squares. The time series of percent valid pixels for each delta is shown in Figure S3.

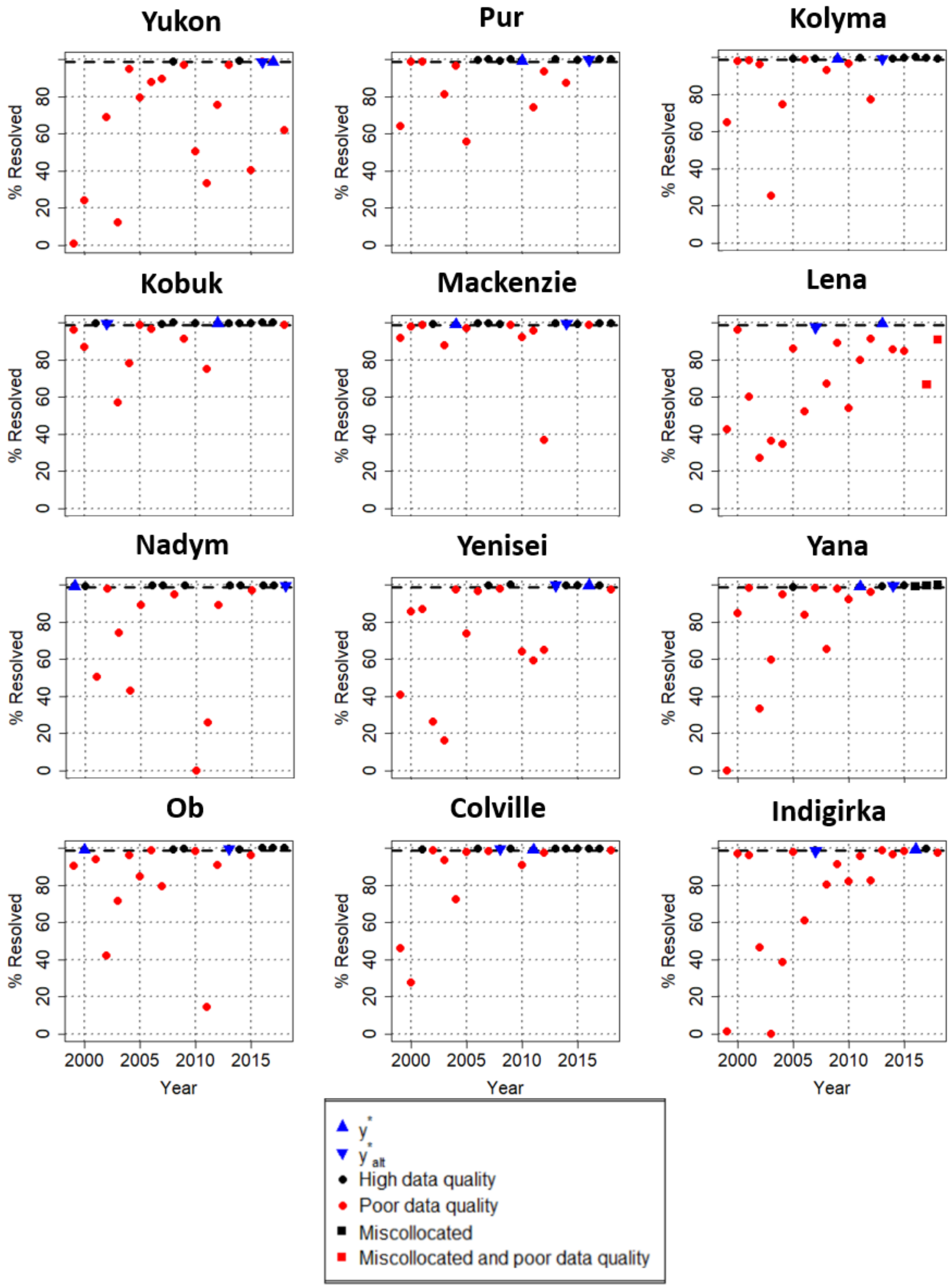

Figure S3. Observational data quality. The percent of pixels resolved in every year on the period of record for the deltas, with symbology the same as in Figure S2.

III. Proportionate growth model

Proportionate growth models, which describe processes where objects grow proportionally to their size with a stochastic growth rate, have seen widespread applications e.g. in modelling micro-organism sizes, income distribution, and city sizes (Crow & Shimizu, 1989; Mitzenmacher 2004). An interesting property of the proportionate growth models is that they result in a lognormal distribution of the size of the objects, with the parameters related to the parameters of the stochastic growth rate. On the basis that the greater thermal inertia of larger lakes results in lake waters remaining unfrozen for longer and maintaining greater lake to soil temperature gradients, we assume that lake growth is proportional to the size of the lake, which has been observed in Alaska (Jones et al., 2011). Then for a lake with radius r_j at the beginning of a time period j of length Δt, its growth rate $\frac{\Delta r_j}{\Delta t}$ is given by Equation (S1).

$$\frac{\Delta r_j}{\Delta t} = r_j k_j. \tag{S1}$$

We can assume that the growth rate k_j at each timestep is an independent and identically distributed random variable characterized by mean γ and variance φ^2, reflecting the variability in water and soil temperature, precipitation, and soil ice content and matrix properties all of which impact lateral heat fluxes. It is easy to show from Equation (S1) that the distribution of the lake radii after some time period t (arising as the sum of the initial lake radius and its subsequent incremental growths Δr_j over the cumulative period of time) will approach a lognormal distribution (Crow & Shimizu, 1989), i.e., $\ln(r) \sim N(\gamma t, \varphi^2 t)$ (see Equation 1 with no lower bound). Assuming a circular shape of the lake, it follows that $\ln(A) = \ln(\pi r^2) \sim N(2\gamma t + \ln(\pi), 4\varphi^2 t) = N(\nu, \beta^2)$, i.e. lake areas are also lognormally distributed with parameters, ν and β^2, and similarly for the volume. A similar model was proposed by Victorov et al. (2019) for thermokarst lakes although empirical testing did not reveal ubiquity of the lognormal size distribution likely due to the mixing of lakes and wetlands in the studied domains.

IV. Fitted distribution parameters and climate trends for lakes, wetlands, and waterbodies

This section contains tables and plots of the fitted distributions and climate trends for lakes, wetlands, and all waterbodies in the reference and alternative reference years. The fitted distribution parameters of lakes and wetlands for a range of waterbody occurrence index thresholds θ used to classify waterbodies extracted in y^* are in Tables (S1 to S3), lake and wetland distribution properties for waterbodies extracted in an alternative reference year y^*_{alt} in Table (S4), the fitted lognormal distribution parameters for waterbody sizes extracted in y^* in Table (S5), the plots of fitted distributions and climate trends of lakes and wetlands extracted in y^*_{alt} (Figures S4 and S5), fitted distributions and climate trends of waterbody sizes extracted in y^* (Figure S6) and boxplots of the waterbody, wetland, and lake size distributions extracted in y^* in Figure (S7).

Table S1. Properties of lake and wetland size distributions at occurrence index threshold θ = 0.85. For each delta, the fitted lognormal parameters ν and β, the number of lakes, N_{Lake}, the p-value (p_{lake}) from a Lilliefors-corrected Kolmogorov Smirnov test (KS test), and $\Delta AIC = AIC_{PL} - AIC_{LN}$ for the lakes, as well as the fitted power law exponent α, fitted minimum lake size x_0, observed maximum wetland size A_{max}, the number of wetlands $N_{wetland}$ in the range [x_0, A_{max}], the p-value ($p_{Wetland}$) from a KS test, and the similarly defined ΔAIC for the wetlands. We report the parameters ν and β in log10 scale rather than in Napierian logarithmic scale (ln) as they are easier to interpret. The fitted distributions which cannot be rejected at the 5% significance level **(p > 0.05) are bolded**.

Delta	N_{Lake}	ν [-]	β [-]	p_{Lake}	ΔAIC Lakes	$N_{Wetland}$ (above x_0)	x_0 [10^5 m^2]	A_{max} [10^5 m^2]	α [-]	$p_{Wetland}$	ΔAIC Wetlands
Yukon	1,511	3.87	0.80	**0.278**	187	401	0.135	2.835	2.55	**0.052**	5.44
Kobuk	1,272	4.40	0.82	**0.688**	328	196	0.09	3.924	2.30	**0.105**	2.88
Nadym	866	4.46	0.70	**0.404**	283	1,005	0.144	52.092	1.91	**0.143**	8.57
Ob	1,567	4.32	0.82	**0.843**	364	940	0.054	31.428	1.77	**0.306**	-1.69
Pur	2,407	4.24	0.75	0.008	537	556	0.117	21.411	1.81	**0.289**	0.48
Mackenzie	20,318	4.37	0.75	0.025	5,517	1,404	0.189	30.168	2.39	**0.636**	-1.70
Yenisei	4,058	4.62	0.60	0.038	2,099	1,028	0.153	10.620	2.47	0.049	6.72
Colville	338	4.57	0.79	**0.326**	111	105	0.162	7.731	2.30	**0.532**	-1.85
Kolyma	3,084	4.19	0.82	**0.283**	595	555	0.135	14.202	2.29	**0.576**	-2.01
Lena	11,265	4.49	0.74	0.008	,3674	1,353	0.477	27.783	2.63	**0.253**	-1.98
Yana	10,297	4.21	0.88	**0.403**	1,949	1,563	0.144	37.872	2.07	**0.511**	-0.77
Indigirka	4,875	3.91	1.08	**0.162**	593	1,830	0.099	42.930	1.91	**0.540**	0.96

Table S2. Properties of lake and wetland size distributions at occurrence index threshold θ = 0.80. Same as Table S1 but with waterbody classification threshold $\theta = 0.8$. Bolded p-values refer to distributions which cannot be rejected at the 5% significance level.

Delta	N_{Lake}	ν [-]	β [-]	p_{Lake}	ΔAIC *Lakes*	$N_{Wetland}$ (above x_0)	x_0 [10^5 m^2]	A_{max} [10^5 m^2]	α [-]	$p_{Wetland}$	ΔAIC *Wetlands*
Yukon	1,829	3.80	0.79	**0.137**	205	252	0.126	1.863	2.74	**0.117**	-0.81
Kobuk	1,417	4.22	0.87	**0.663**	274	185	0.054	3.924	2.22	**0.709**	0.91
Nadym	1,311	4.31	0.73	**0.645**	328	1,452	0.063	42.876	1.89	0.019	10.61
Ob	1,773	4.17	0.88	**0.825**	318	734	0.054	21.483	1.82	**0.298**	-1.58
Pur	2,796	4.07	0.81	**0.168**	462	784	0.054	21.411	1.85	0.001	-1.88
Mackenzie	22,495	4.24	0.79	0.016	4816	1,019	0.153	19.620	2.37	**0.824**	-2.01
Yenisei	4,889	4.50	0.62	0.023	2001	765	0.126	9.090	2.65	**0.773**	-1.66
Colville	407	4.38	0.84	**0.215**	98	109	0.108	7.731	2.22	**0.720**	-1.99
Kolyma	3,613	3.98	0.87	**0.435**	508	692	0.072	14.202	2.31	**0.995**	-2.20
Lena	14,156	4.35	0.76	0.047	3644	637	0.540	19.008	2.63	**0.481**	-2.00
Yana	11,567	4.08	0.91	**0.756**	1827	2,015	0.072	12.789	2.10	**0.251**	-1.98
Indigirka	5,440	3.74	1.12	**0.062**	547	1,433	0.099	25.299	1.91	**0.879**	-0.70

Table S3. Properties of lake and wetland size distributions at occurrence index threshold θ = 0.9. Same as Table S1 but with waterbody classification threshold $\theta = 0.9$. Bolded p-values refer to distributions which cannot be rejected at the 5% significance level.

Delta	N_{Lake}	ν [-]	β [-]	p_{Lake}	ΔAIC *Lakes*	$N_{Wetland}$ (above x_0)	x_0 [10^5 m^2]	A_{max} [10^5 m^2]	α [-]	$p_{Wetland}$	ΔAIC *Wetlands*
Yukon	1,118	3.95	0.81	**0.279**	152	185	0.369	15.993	2.69	**0.985**	-1.73
Kobuk	1,022	4.62	0.76	**0.827**	384	100	0.333	4.311	2.76	**0.597**	-2.00
Nadym	433	4.61	0.71	**0.395**	172	262	1.008	52.092	2.23	**0.610**	-0.15
Ob	1,275	4.50	0.75	**0.677**	410	1,232	0.054	43.704	1.75	**0.641**	2.14
Pur	1,753	4.47	0.69	0.025	600	1,356	0.081	23.697	1.85	**0.816**	2.13
Mackenzie	16,395	4.55	0.70	**0.091**	6,130	2,941	0.198	30.168	2.30	0.000	16.69
Yenisei	2,883	4.76	0.58	**0.625**	1,905	497	0.486	10.620	2.73	**0.281**	1.65
Colville	248	4.77	0.78	**0.382**	107	167	0.162	7.731	2.22	**0.255**	0.85
Kolyma	2,218	4.42	0.79	**0.730**	610	352	0.378	14.202	2.37	**0.946**	-1.91
Lena	7,438	4.67	0.73	0.000	3,151	2,369	0.495	27.783	2.43	**0.339**	6.64
Yana	8,286	4.34	0.86	0.016	1,884	2,806	0.144	37.872	1.96	0.000	34.68
Indigirka	3,973	4.06	1.08	**0.264**	564	1,113	0.270	73.431	1.93	**0.276**	1.19

Table S4. Properties of lake and wetland size distributions for waterbody extents identified in an alternative reference year. Same as Table S1 but for waterbody extent identified in an alternative reference year, y^*_{alt}, with close to average water cover, and using an occurrence index threshold $\theta = 0.85$. Bolded p-values refer to distributions which cannot be rejected at the 5% significance level.

Delta	N_{Lake}	ν [-]	β [-]	p_{Lake}	ΔAIC Lakes	$N_{Wetland}$ (above x_0)	x_0 [10^5 m^2]	A_{max} [10^5 m^2]	α [-]	$p_{Wetland}$	ΔAIC Wetlands
Yukon	1,340	3.90	0.81	**0.767**	171	961	0.081	3.357	2.10	0.003	36.06
Kobuk	1,421	4.28	0.83	**0.302**	305	196	0.054	2.025	2.25	**0.517**	2.13
Nadym	867	4.40	0.72	**0.396**	255	1,358	0.108	50.175	1.81	0.001	28.09
Ob	1,440	4.49	0.78	0.007	439	361	0.288	8.766	2.45	**0.238**	2.63
Pur	2,132	4.58	0.63	0.002	968	404	0.234	15.867	2.59	**0.106**	-1.03
Mackenzie	18,256	4.46	0.73	**0.080**	5,808	2,084	0.189	28.251	2.41	0.001	15.53
Yenisei	4,040	4.62	0.60	**0.072**	2,094	344	0.324	8.127	2.84	**0.385**	-0.80
Colville	441	4.25	0.88	**0.312**	86	140	0.072	2.934	2.20	**0.687**	-1.46
Kolyma	2,321	4.38	0.80	**0.511**	596	988	0.153	15.183	2.10	0.029	12.55
Lena	12,467	4.37	0.77	**0.059**	3,299	1,633	0.324	48.402	2.34	**0.360**	-1.99
Yana	10,145	4.31	0.84	**0.331**	2,267	2,011	0.126	16.470	2.21	**0.090**	7.12
Indigirka	5,892	3.90	1.05	**0.197**	714	866	0.117	23.193	2.31	**0.052**	-2.01

Table S5. Lognormal waterbody size distribution parameters. Fitted lognormal parameters ν and β, for the waterbody size distribution in the reference year y^*, the number of waterbodies, $N_{waterbody}$, KS test p-values ($p_{Waterbody}$) used to evaluate the goodness of fit, and the ΔAIC as defined above (see caption of Table S1). Bolded p-values refer to distributions which cannot be rejected at the 5% significance level.

Delta	$N_{Waterbody}$	ν [-]	β [-]	$p_{Waterbody}$	ΔAIC Waterbodies
Yukon	2,610	2.97	0.97	**0.350**	103
Kobuk	1,602	3.92	0.97	**0.130**	201
Nadym	2,945	3.26	1.01	**0.417**	169
Ob	2,507	3.51	1.08	0.012	196
Pur	3,580	3.63	0.95	**0.251**	315
Mackenzie	25,995	3.96	0.88	0.000	3,543
Yenisei	6,981	3.97	0.81	0.005	991
Colville	606	3.50	1.09	**0.417**	45
Kolyma	4,557	3.35	1.04	**0.674**	296
Lena	25,604	3.20	1.06	0.000	1,421
Yana	14,283	3.53	1.06	0.000	1,151
Indigirka	7,807	2.70	1.36	0.043	319

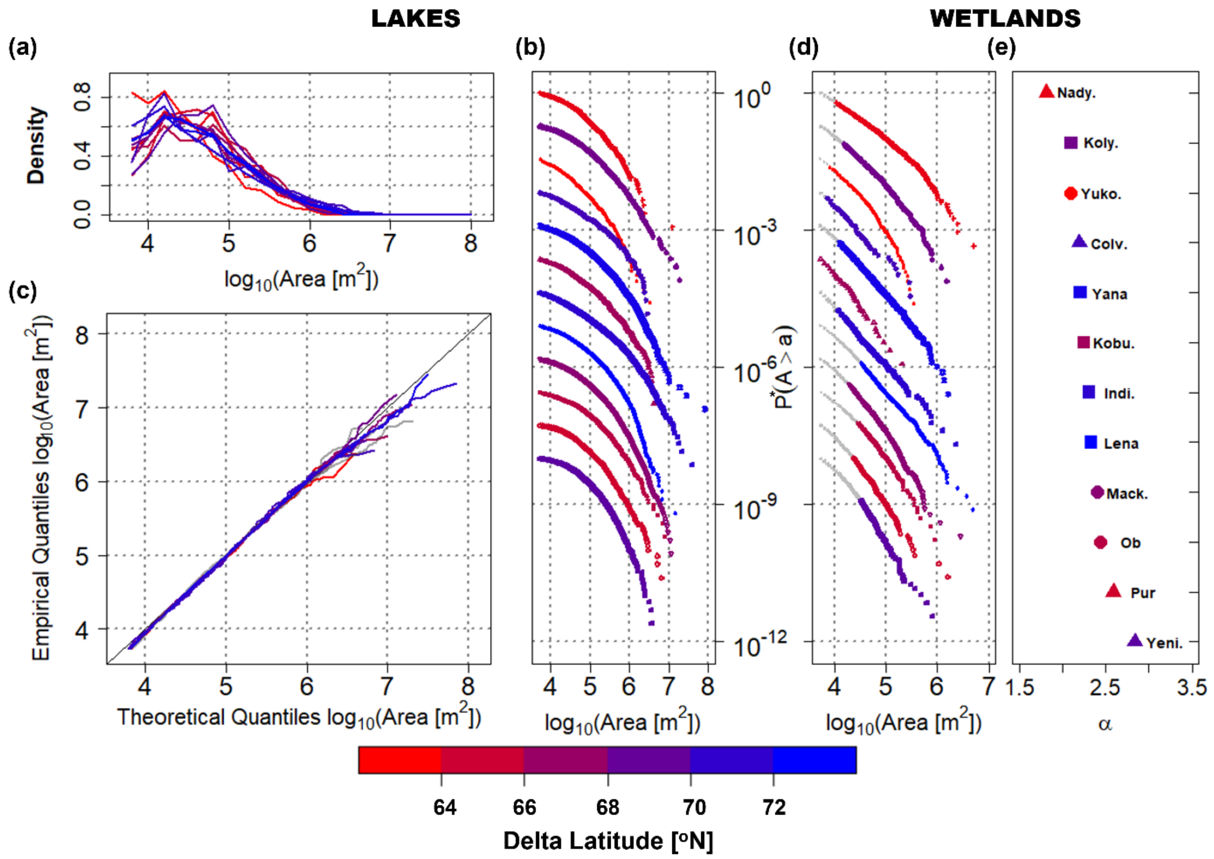

Figure S4. Lake and wetland size distributions extracted in an alternative reference year. Same as Figure 3 but for waterbody extents identified in an alternative reference year, y^*_{alt}, for all 12 deltas. A truncated lognormal distribution is significant for the lake area distribution at the 5% significance level (KS test) for 10 deltas. The KS test does not reject a power law for the upper tails of the wetland size distributions in 8 out of 12 deltas at a 5% significance level.

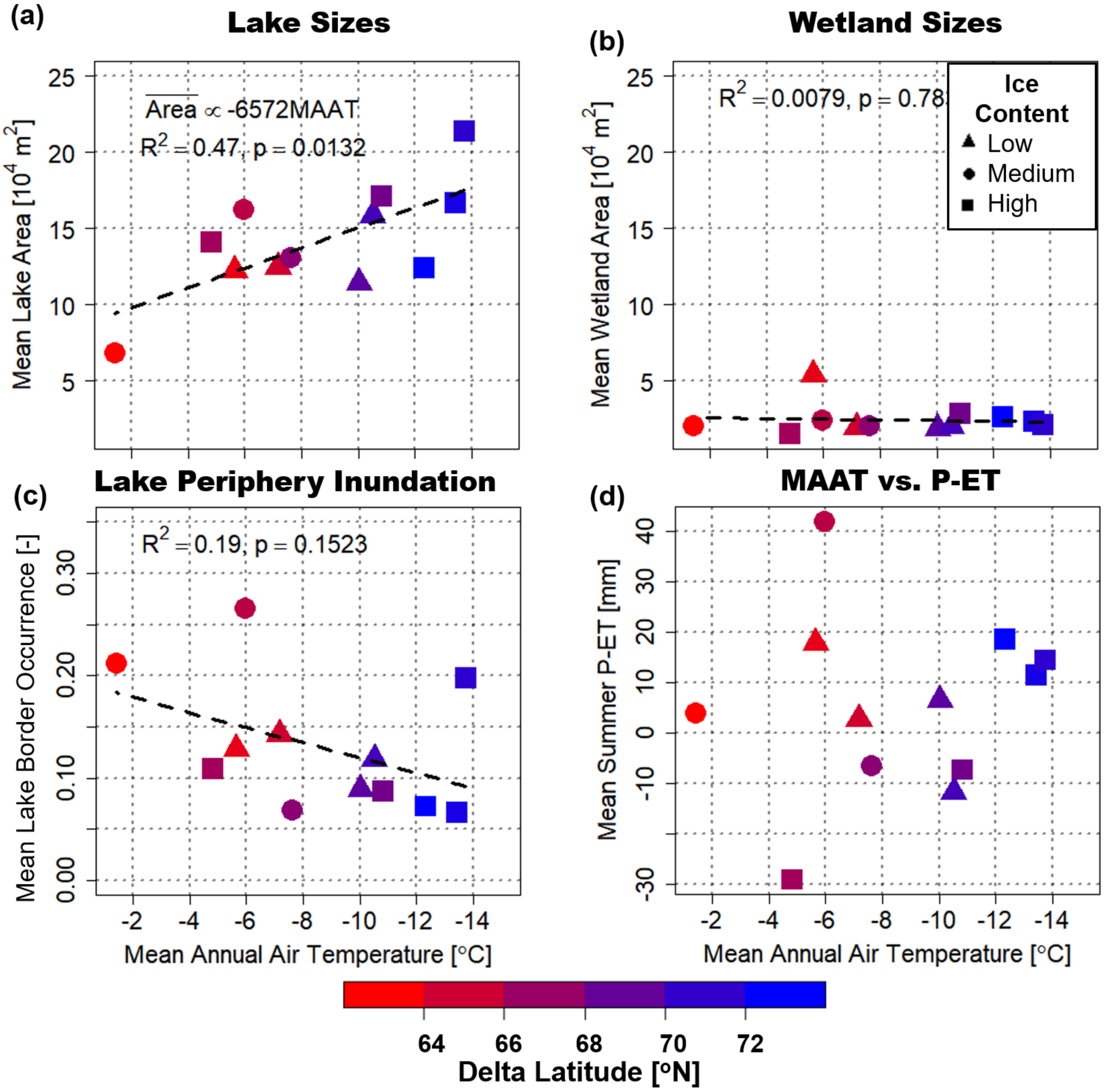

Figure S5. Climate trends for lakes and wetlands extracted in an alternative reference year. (a-c) are the same as Figures 4a, 4b, and 4e, but for waterbody extents identified in an alternative reference year, y^*_{alt}, for all 12 deltas. In (a) the trend between MAAT and mean lake area has bootstrap p = 0.0116 and a Spearman rank correlation of -0.59 (p = 0.0384). In (c), the presence of two large outliers (Ob and Indigirka) renders the trend non-significant. Excluding them to evaluate the relationship among the rest of deltas yields a significant trend (R^2 = 0.66, p = 0.005), supporting a possible relationship. (d) Scatterplot of 2000-2016 mean June to July precipitation minus evapotranspiration (P-ET) over the deltas versus MAAT (Bromwich et al., 2018), indicating vertical hydrologic budget is unrelated to differences in MAAT (R^2 = 0.013) and therefore does not explain the relationship in (c) or in Figure 4e.

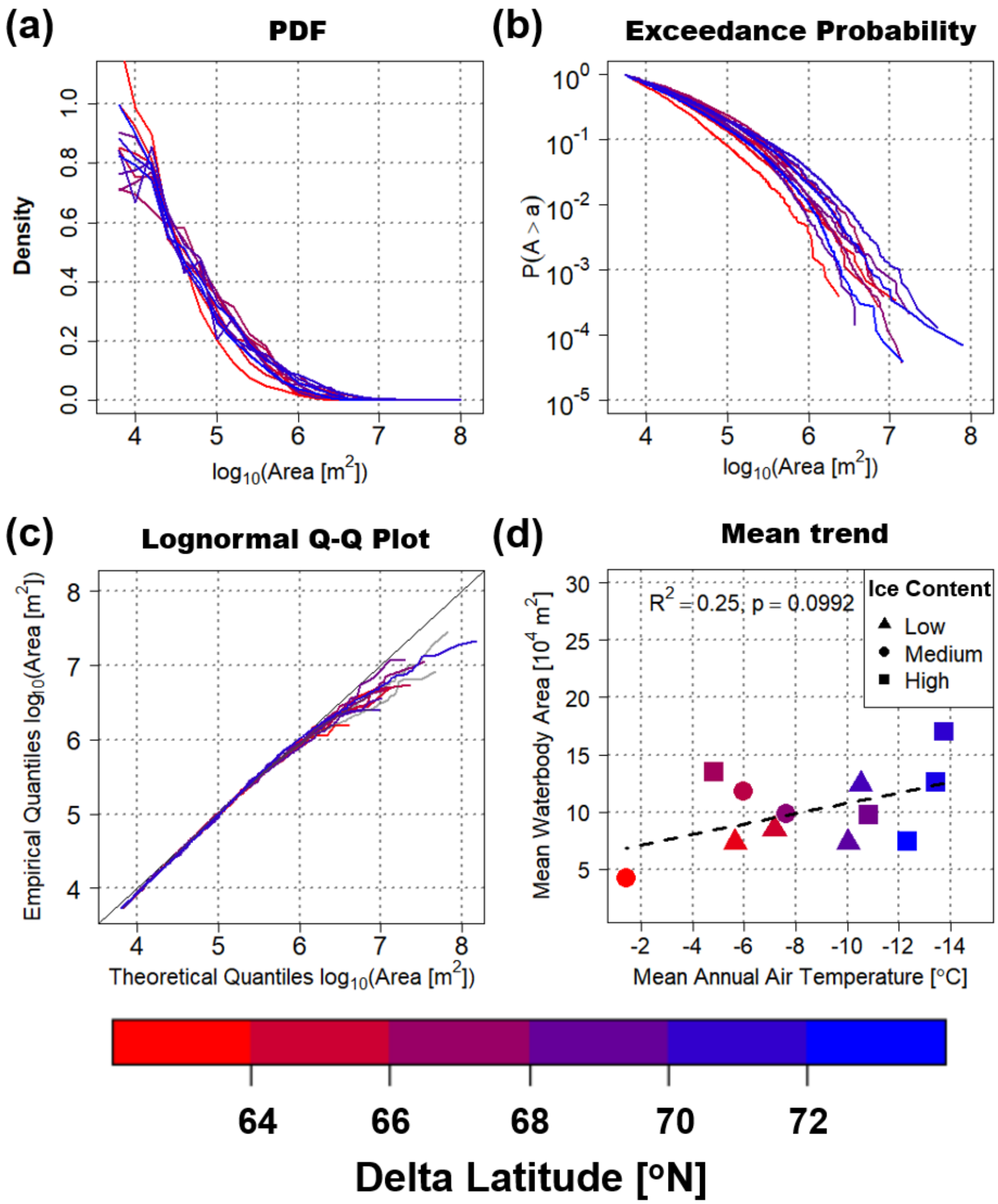

Figure S6. Waterbody size distributions and goodness of fit: (a) The PDF and (b) exceedance probability curves of the waterbody (lake and wetlands combined) size distributions extracted in the reference year y^*, for all 12 deltas. (c) Q-Q plots of the lognormal distribution fit to the waterbody sizes, for all 12 deltas, with the fitted distributions which are not statistically significant at the 5% significance level (KS test) in grey. (d) Scatterplot of mean waterbody area and MAAT, with delta ice content indicated by point symbol, shows no statistically significant linear relationship between the two

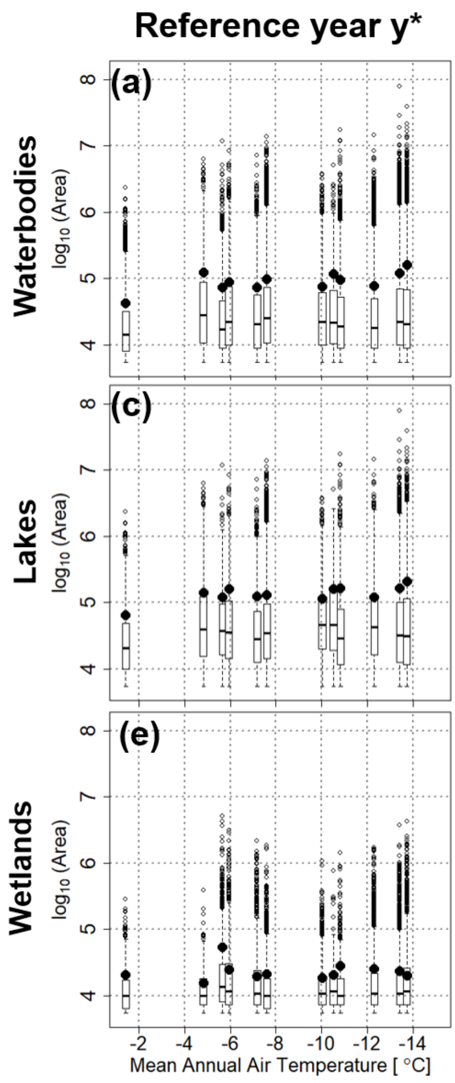

Figure S7. Waterbody, lake, and wetland size distribution boxplots. (a-c) Boxplots of the size distribution for all waterbodies (a), lakes (b), and wetlands (c), with boxes representing the interquartile range, whiskers 1.5x the interquartile range, horizontal lines the sample median, and black dots the sample mean.

V. Model identification for small samples of power-law distributed data.

Although the hypothesis of a power law distribution for the wetland sizes could not be rejected in the majority of deltas (Tables S1 to S4), it is reasonable to ask whether the alternative hypothesis of a truncated lognormal (LN) distribution for wetland sizes could also be statistically acceptable, or even be a better fit in some cases for the wetland sizes. In principle, comparison of the fitted power law and LN distributions can be performed using the Akaike Information Criterion (AIC) test (Burnham and Anderson, 2004) or the likelihood ratio test (Clauset et al., 2009). However, the small sample size available for this testing (~200 to 2,000 wetlands in our case) introduces challenges in robustly differentiating the power law distribution from the LN distribution. To gain quantitative insight into this problem, we performed a simulation of power law distributed data and used the AIC test to determine whether indeed the power law emerges as a better candidate distribution than the LN distribution. Specifically, we simulated synthetic power-law data using the procedure outlined in Clauset et al. (2009) with parameters $\alpha = 2.07$, $x_0 = 14{,}400$ m^2, and $N = 1{,}563$, i.e., the fitted parameters and sample length of the Yana wetland sizes extracted in y^* at a threshold of $\theta = 0.85$ (Table S1) and repeated this exercise for a large number of simulations ($M = 1000$). For each simulated data set i we used the same fitting procedures as for the observed wetland sizes, i.e., fitted a power-law distribution by estimating x_0^i and α^i and then fitted a truncated lognormal with $x_{min} = x_0^i$ and estimating ν^i and β^i. We then computed the AIC difference $\Delta AIC^i = AIC_{PL}^i - AIC_{LN}^i$, where a positive ΔAIC^i indicates the lognormal is a better fit and a negative value indicates the power law is a better fit, and examined the PDF of ΔAIC over the 1000 simulations (Figure S8a). We also examined the likelihood that the model rejected by the AIC test is a better candidate for the data by computing $e^{-\frac{|\Delta AIC_i|}{2}}$ (Figure S8b; Burnham and Anderson, 2004).

The PDF of ΔAIC is centered at -2, indicating that the power law is a better fit overall, however this is not a large enough difference to reject the alternative hypothesis of the LN (Burnham and Anderson, 2004). Moreover, in approximately 9% of the cases ΔAIC is larger than zero, and therefore, the LN would be considered a better fit. It has also been shown that other statistical tests to compare competing hypotheses, such as the related likelihood ratio test, cannot distinguish between power-law and LN distributions for sample sizes below 2,000 (Figure 6 in Clauset et al., 2009). Based on these experiments, it is evident that we cannot solely rely on statistical tests to decide which distribution is better representing heavy-tailed data with sample sizes below 2,000. Therefore, physical reasoning along with the interpretation of the fitted parameters are necessary to establish which distribution might be more suitable and interpretable for a given data set.

Looking back to the wetland size distributions which have statistically significant power law fits (Tables S1 to S4), $\Delta AIC = AIC_{PL} - AIC_{LN}$ tends to fall within the range of -2 to 2, which are values typically observed for truly power-law distributed data with the given sample sizes. Moreover, in other environments, wetlands identified by inundating rough topography show power-law slopes in a similar range (Le and Kumar, 2014; Bertassello et al., 2018) to what is reported herein (See Figure 3 and Tables S1 to S3), suggesting that ephemeral wetlands on arctic deltas may be governed by similar forming processes as wetlands elsewhere, giving rise to

emergent power law size distributions. We also found that the parameters of the fitted LN distributions typically corresponded to $\nu \ll 0$ in the log10 scale and ended up fitting the data to a negligible (much smaller than 1%) fraction of the upper tail of an LN distribution. Therefore, we assert that the power law distribution is a more physically and statistically meaningful descriptor of the wetland size distribution compared with the LN. For completeness, we also tested the alternative hypothesis of a power law distribution with $x_0 = 5{,}400\ \mathrm{m}^2$ for the lake sizes (Tables S1 to S4) and waterbody sizes (Table S5) and report the AIC test results with the same notation. We found that in all cases the LN distribution for lake sizes was a significantly better fit than a power law and therefore a better descriptor of the lake size distribution.

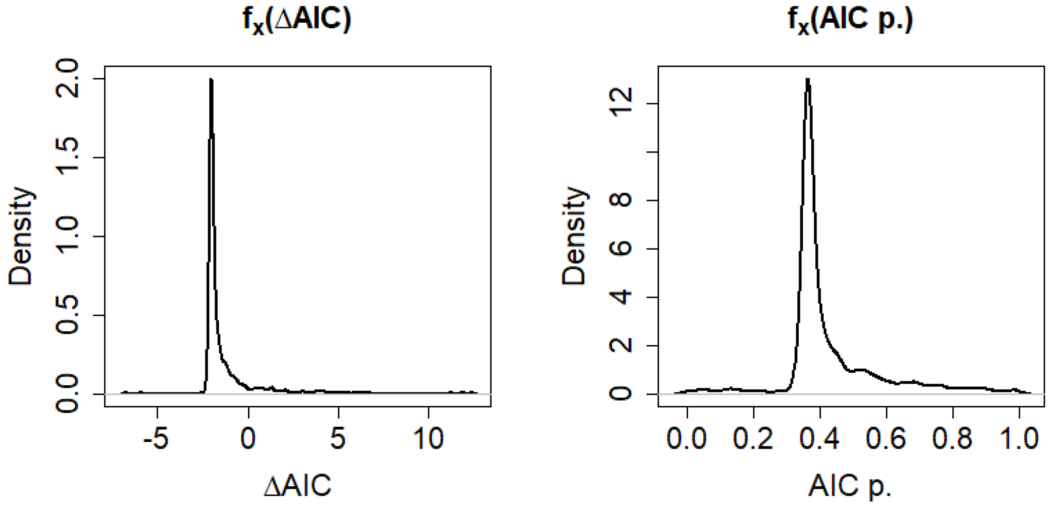

Figure S8. AIC test results to distinguish between power law and lognormal distributions fitted to simulated power law data. (a, b) The probability distribution of $\Delta AIC^i = AIC^i_{PL} - AIC^i_{LN}$ for 1,000 simulated power law distributions with $\alpha = 2.07,\ x_0 = 14{,}400\ \mathrm{m}^2$, and $N = 1{,}563$ (a) along with the likelihood, i.e. $e^{-\frac{|\Delta AIC_i|}{2}}$, that the model rejected by the AIC test is a better candidate for the data (b). Although the underlying data are truly power law distributed in every single case, both test statistic distributions are centered at values ($\Delta AIC = -2, p = e^{-1}$) that preclude inferring that the data in all 1000 simulations are indeed power law distributed.

VI. Relationships between the first three conditional moments of waterbody sizes

Muster et al. (2019) analyzed 30 regional size distributions of ponds and lakes from the circum-Arctic Permafrost Region Pond and Lake (PeRL) database, and found a linear relationship between the sample mean and the variance, and a hyperbolic relationship between the sample mean and the skewness coefficient of the empirical distributions when estimating these moments over a bounded range, e.g. a lower bound a and an upper bound b, also called the conditional sample moments. They also found that the statistical moments of waterbody sizes identified by inundating a digital elevation model exhibited similar relationships, and therefore determined that pond and lake sizes likely reflect landscape inundation level, rather than reflecting temperature driven growth due to climate. We compared the conditional sample moments of the 30 PeRL regional size distributions and the conditional moments of the fitted LN distributions to the lake sizes of the 12 arctic deltas to investigate if they displayed similar scaling relationships. In Muster et al. (2019) the bounds to compute the conditional sample moments used were a = 100 m^2, the minimum reliable lake size from PeRL, and b = 10^6 m^2 an upper bound to account for poor sample size for large lakes. We used for both the PeRL regions and the 12 deltas a = 5.4·10^3 m^2, the minimum reliable lake size estimate in our study and b = 10^6 m^2, the same upper bound used in their study. As the relationships between the conditional sample moments computed from the fitted LN size distribution arising from proportionate growth are indistinguishable from the sample moments of the PeRL database (Figure S9), we caution that such relationships cannot be used to differentiate between probability distributions and the different mechanisms underlying wetland (inundation) and lake (proportionate growth) formation.

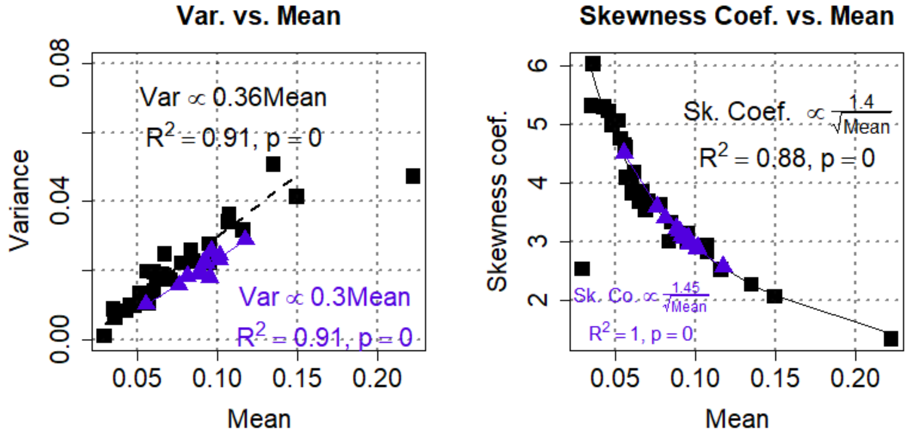

Figure S9. Lake size conditional moments of the fitted LN PDFs compared with PeRL lake and pond size sample conditional moment scaling relationships. The conditional mean and conditional variance (a) and the conditional mean and the conditional skewness coefficient (b) of the lakes on our 12 arctic deltas (purple triangles) and lakes and ponds examined in Muster et al. (2019) (black squares). The outlier at (0.23 km^2, 0.05 km^4) was discarded to fit the mean and variance relationship (a) and the outlier at (.01 km^2, 2.2) were discarded to fit the mean and skewness relationship (b) for the PeRL data.
